# Supplementary material for: Learning on tree architectures outperforms a convolutional feedforward network
Source: Sci Rep. 2023 Jan 30;13:962. doi: 10.1038/s41598-023-27986-6 (PMC9886946; doi:10.1038/s41598-023-27986-6)
Supplement: Supplementary file 1 — Supplementary Figures. [file 41598_2023_27986_MOESM1_ESM.pdf]

Learning on tree architectures outperforms a convolutional feedforward network

Yuval Meir[1], Itamar Ben-Noam[1], Yarden Tzach[1], Shiri Hodassman[1] and Ido Kanter[1,2*]

[1]Department of Physics, Bar-Ilan University, Ramat-Gan, 52900, Israel.

[2]Gonda Interdisciplinary Brain Research Center, Bar-Ilan University, Ramat-Gan, 52900, Israel.

[*]Corresponding author email: ido.kanter@biu.ac.il

This PDF file includes:

Figures. S1 and S2

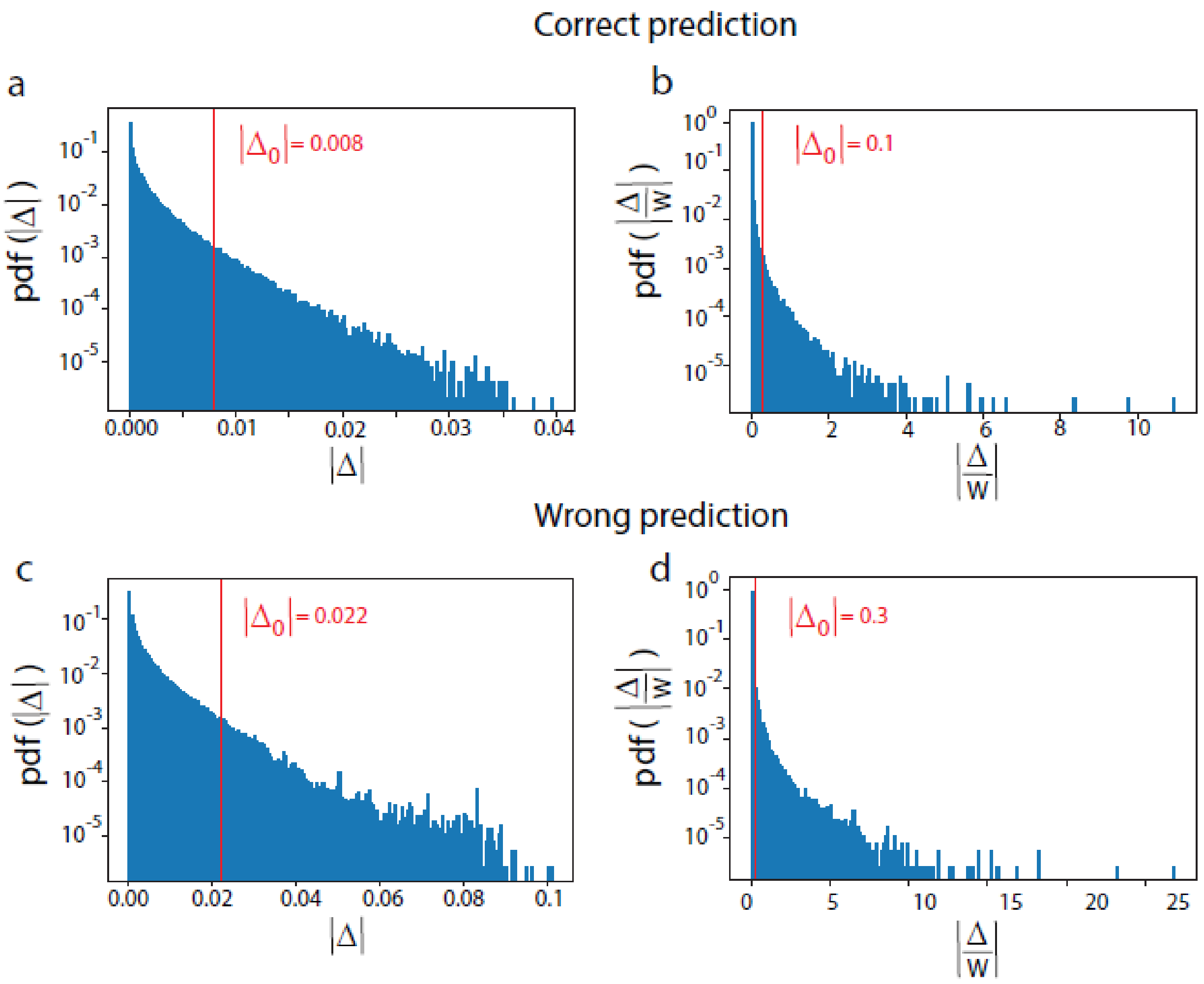

Supplementary Figure S1. Probability density function (pdf) of W^{Conv} gradients, $|\Delta|$, and $|\Delta/W^{Conv}|$ for Tree-3 using ReLU activation function. a, pdf of the absolute value of $|\Delta|$for a test example of with a correct predicting label. The vertical red line stands for Δ_0 (denoted in red) such that the summation of pdf($\Delta < \Delta_0$) ~ 0.97. **b**, Similar to (**a**) for $|\Delta/W^{Conv}|$. **c**, Similar to (**a**) with a wrong predicting label. **d**, Similar to (**b**) with a wrong predicting label. Each one of the histograms consists of 1000 bins. In all panels, the vertical axis is in log-scale.

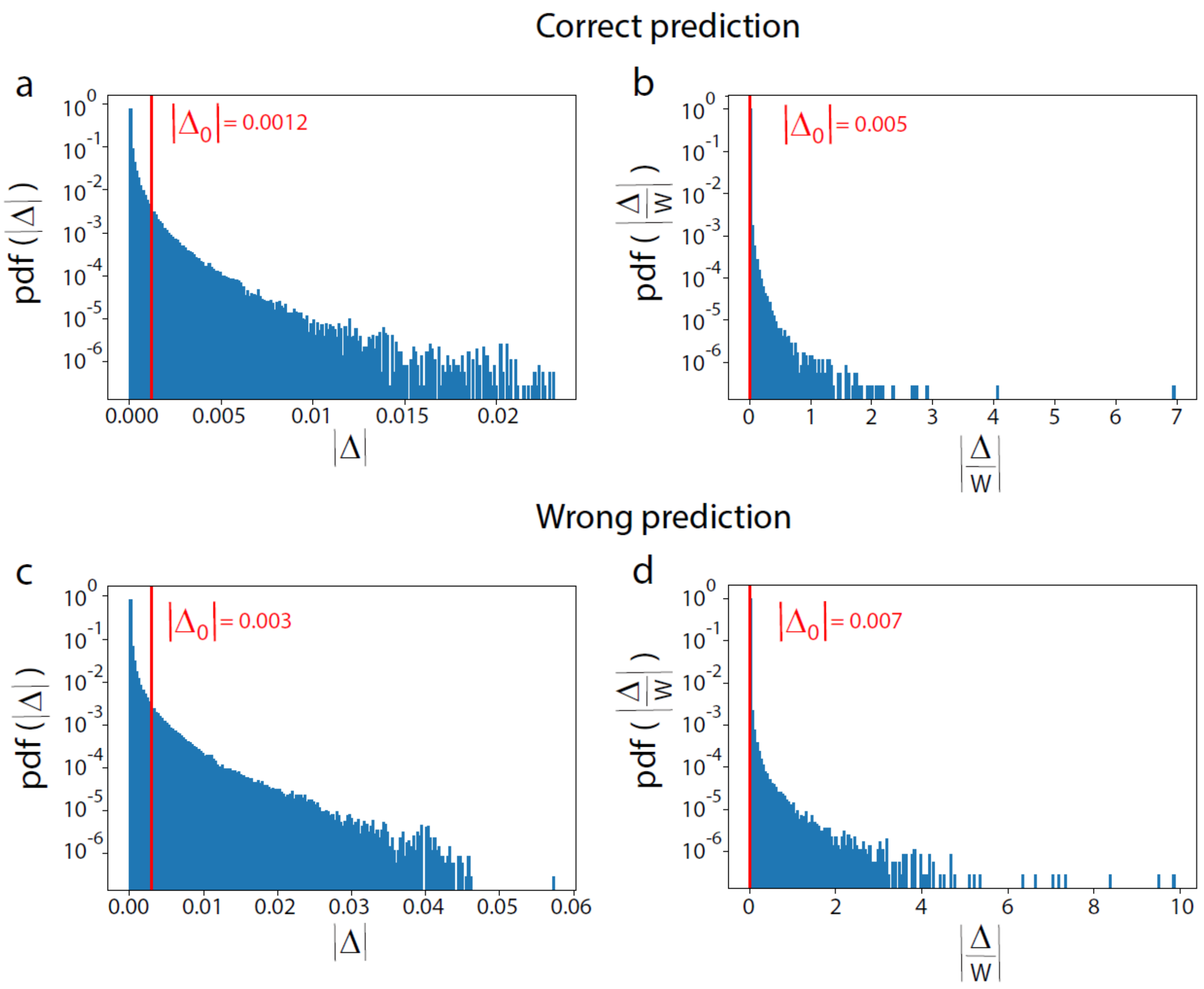

Supplementary Figure S2. Probability density function (pdf) of W^{Conv} gradients, $|\Delta|$, and $|\Delta/W^{Conv}|$ for Tree-3 using Sigmoid activation function. a, pdf of the absolute value of $|\Delta|$ for a test example with a correct predicting label. The vertical red line stands for Δ_0 (denoted in red) such that the summation of pdf($\Delta < \Delta_0$) ~ 0.97. **b**, Similar to (**a**) for$|\Delta/W^{Conv}|$. **c**, Similar to (**a**) with a wrong predicting label. **d**, Similar to (**b**) with a wrong predicting label. Each one of the histograms consists of 1000 bins. In all panels, the vertical axis is in log-scale.
